# Nanomaterial-Mediated RNAi Targeting Chitin Metabolism Genes in MEAM1 Cryptic Species of *Bemisia tabaci* (Hemiptera: Aleyrodidae)

**DOI:** 10.3390/insects17010002

**Published:** 2025-12-19

**Authors:** Dejun Kong, Huarong Gu, Yinglu Gao, Yangnan Hou, Jigang Li

**Affiliations:** 1 School of Life Sciences, Hebei University, Baoding 071002, China; xk143285@163.com (D.K.); 18784009407@163.com (H.G.); gyldeyx_1@163.com (Y.G.); hynn145890@163.com (Y.H.); 2 Key Laboratory of Microbial Diversity Research and Application of Hebei Province, Hebei University, Baoding 071002, China; 3 Engineering Research Center of Ecological Safety and Conservation in Beijing-Tianjin-Hebei (Xiong’an New Area) of MOE, Hebei University, Baoding 071002, China

**Keywords:** chitin metabolism, RNA interference, nanomaterial, *Bemisia tabaci*, pest control

## Abstract

The whitefly *Bemisia tabaci* is a globally invasive pest that threatens crop production through feeding and virus transmission. In this study, we identified genes encoding enzymes in the chitin metabolism pathway of *B. tabaci*—β-N-acetylglucosaminidase (BtNAG), N-acetylglucosamine kinase (BtNAGK), phosphoacetylglucosamine mutase (BtPAGM), UDP-N-acetylglucosamine pyrophosphorylase (BtUAP), and glucosamine-6-phosphate N-acetyltransferase (BtGNA)—using bioinformatic analysis. Quantitative reverse-transcription PCR (RT-qPCR) analyses revealed distinct stage-specific expression patterns for these genes. We used the nanomaterial star polycation (SPc) to deliver gene-specific double-stranded RNA (dsRNA) targeting these genes to fourth instar *B. tabaci* nymphs, which resulted in significant mortality and developmental defects upon gene silencing. Notably, the fusion dsRNA targeting three genes—*BtNAG1*, *BtNAGK*, and *BtUAP*—achieved approximately 80% nymph mortality, 70% inhibition of adult emergence, and an earlier onset of gene silencing. These findings provide evidence that nanomaterial-assisted delivery of dsRNA can significantly enhance RNAi effects in hemipteran pests and that dsRNA targeting chitin metabolic genes may be an effective strategy for RNAi-based control of *B. tabaci*.

## Simple Summary

Chitin metabolism plays a critical role in the growth and development of insects and offers potential targets for RNA interference (RNAi)-based pest management. We identified five functional genes in the chitin metabolic pathway of *Bemisia tabaci* and analyzed their expression patterns in various developmental stages. Using a nanocarrier-based dsRNA delivery system, we achieved efficient gene silencing of genes in the chitin metabolism pathway, resulting in an increase in nymph mortality and a marked reduction in adult emergence rates. Moreover, nymphs exposed to multi-target dsRNA showed an earlier onset of gene silencing than those treated with single-target dsRNAs.

## 1. Introduction

The whitefly *Bemisia tabaci* (Hemiptera: Aleyrodidae) is a globally destructive agricultural pest characterized by its invasiveness, wide distribution, and broad genetic diversity [1]. This species includes over 40 biotypes, among which the Middle East-Asia Minor 1 (MEAM1, or B biotype) and the Mediterranean (MED, or Q biotype) biotypes are the most damaging and widely distributed [2,3]. It can infest over 1000 plant species, affecting crops ranging from fruits and vegetables (such as beans, cucumber, and tomato) to cotton and other crops. It primarily harms plants by directly feeding on phloem sap and indirectly transmitting plant viruses, leading to reduced crop yields and significant economic losses [1,4,5]. Additionally, *B. tabaci* adults excrete honeydew while feeding, which covers plant surfaces and promotes the growth of black sooty mold, further reducing the production and economic value of crops [1].

Control of *B. tabaci* has long been a challenge due to its adaptability and resistance to various management strategies. Numerous studies have shown that the overuse of insecticides significantly reduces non-pest insect populations, including beneficial insects and natural enemies [6]. Moreover, this overuse accelerates the development of resistance in *B. tabaci* against most insecticides [7]. The expression of *Bacillus thuringiensis* delta-endotoxin insecticidal proteins in transgenic crops has proven ineffective against whiteflies and other sap-sucking pests as these pests are insensitive to these toxins [8]. Other approaches, such as biological control, have significantly reduced whitefly populations, though applying pesticides before releasing biocontrol agents is recommended to achieve better results [9].

Chitin, the second most abundant polysaccharide on Earth, is a principal constituent of the insect exoskeleton, peritrophic matrix, and wing buds. It plays crucial roles in maintaining the specific shape of insects, resisting mechanical damage, and protecting against pathogens [10,11,12,13,14]. Throughout the entire life cycle of an insect—from egg to adult—the biosynthesis, degradation, and modification of chitin occur regularly. Chitin biosynthesis in insects is accomplished through the sequential activities of eight enzymes. The first five enzymes, Trehalase (TRE), Hexokinase (HK), Glucose-6-phosphate isomerase (G6PI), Glutamine:fructose-6-phosphate aminotransferase (GFAT), and Glucosamine 6-phosphate N-acetyltransferase (GNA), catalyze the generation of amino sugar N-acetylglucosamine (GlcNAc) from trehalose. The activation of GlcNAc to UDP-GlcNAc is then catalyzed by phosphoacetylglucosamine mutase (PAGM) and UDP-N-acetylglucosamine pyrophosphorylase (UAP). Finally, the polymerization of UDP-GlcNAc monomer into chitin is catalyzed by the membrane-integral chitin synthase (CHS) [10]. Chitin degradation can proceed via two biochemical pathways. In one pathway, chitin is hydrolyzed by chitinase (CHT) to yield oligomeric N-acetylglucosamine (GlcNAc) units, which are subsequently cleaved by β-N-acetylglucosaminidase (NAG) to release GlcNAc monomers. The action of chitin deacetylases (CDAs) can be viewed either as an alternative degradation pathway or as a chitin modification step. CDAs deacetylate chitin to form chitosan, which is subsequently degraded by chitosanase and glucosaminidase to produce glucosamine (GlcN) monomers [15].

Although chitin metabolism presents many potential control targets, currently commercialized pesticides are primarily restricted to inhibiting chitin synthase. These chitin synthesis inhibitors (CSIs) include benzoylphenyl ureas (BPUs) like diflubenzuron, and non-BPU growth regulators, such as buprofezin and cyromazine. Like other chemical pesticides, the excessive use of some CSIs has led to pest resistance, and the environmental toxicity of some CSIs to aquatic invertebrates is non-negligible [14].

RNA interference (RNAi) is a highly conserved mechanism among eukaryotes. In this process, double-stranded RNA (dsRNA), either exogenous dsRNA that is ingested or dsRNA that is endogenously expressed, works in concert with various proteins and enzymes to trigger the degradation of homologous endogenous mRNA, leading to the silencing of target genes [16]. RNAi has been applied in various pest management strategies, such as reducing pest resistance to insecticides or disrupting the developmental processes of pests [17]. Significant advancements have been made in employing RNAi strategies to manage cotton bollworms (*Helicoverpa armigera*) and corn rootworms (*Diabrotica virgifera virgifera*) [18,19].

Nanomaterials is a promising tool for enhancing the delivery of dsRNA in RNAi. These nanoscale particles generally have positively charged or anion-exchangeable surfaces, low toxicity, and high biocompatibility. Currently, a variety of nanomaterials have been tested for their effectiveness in dsRNA delivery in insects. Such materials include chitosan, liposomes, SPcs, carbon quantum dots (CQDs), layered double hydroxides (LDHs), guanylated polymers (GNPs), branched amphiphilic peptide capsules (BAPCs), and cell-penetrating peptides (CPPs) [20]. Among these materials, SPcs are particularly attractive due to their cost-effectiveness, facile synthesis, ability to stabilize dsRNA, and capacity to promote cellular uptake of dsRNA [21].

Because chitin is absent in vertebrates and higher plants, enzymes in the chitin metabolic pathway are potential, environmentally safe, and highly efficient targets for pesticide development [11,14,22]. To date, RNAi experiments targeting chitin metabolic genes have only rarely been reported [23]. Therefore, there is an urgent need to develop pest control strategies using RNAi to target chitin metabolism. In this study, bioinformatic tools were used to identify the genes in the chitin metabolic pathway. Based on the temporal expression patterns of these genes, we performed RNAi on *B. tabaci* nymphs to evaluate the efficacy of the nanomaterial SPc in dsRNA delivery and to assess the potential of RNAi targeting chitin-metabolism genes for *B. tabaci* control.

## 2. Materials and Methods

### 2.1. Insects

Adults of the *B. tabaci* cryptic species MEAM1 were collected and identified from the Hebei University campus (115.564881° E, 38.881407° N, Lianchi District, Wusidong Road, Baoding, China) in October 2022. Since then, the *B. tabaci* strain has been maintained on upland cotton seedlings within insect-proof rearing cages. The rearing conditions were maintained at a temperature of 26 ± 1 °C, relative humidity of 60 ± 10%, and a photoperiod of 16 h of light and 8 h of darkness. The purity of the *B. tabaci* strain was monitored every 3 to 5 generations using polymerase chain reaction (PCR) and sequencing to analyze the mitochondrial cytochrome oxidase I (mtDNA COI) gene sequence [24,25,26].

### 2.2. Bioinformatic Analysis of Genes

The reference genome data for biotype B (MEAM1) of *B. tabaci* was retrieved from the NCBI database (https://www.ncbi.nlm.nih.gov/datasets/genome/GCF_001854935.1/ (accessed on 15 October 2023)). The software eggNOG-Mapper v2 (http://eggnog-mapper.embl.de/ (accessed on 15 October 2023)) was utilized for gene function annotation. The KEGG database (http://www.genome.jp/kegg/ (accessed on 15 October 2023)) was employed to map the chitin metabolism pathways. The protein molecular weights and isoelectric points were calculated and predicted using the ExPASy Proteomics Server (http://expasy.org/ (accessed on 15 October 2023)). The subcellular localization of proteins was predicted through Cell-PLoc (http://www.csbio.sjtu.edu.cn/bioinf/Cell-PLoc/ (accessed on 15 October 2023)). The hydrophilic/hydrophobic characteristics of proteins were analyzed using Protscale online software (https://web.expasy.org/protscale/ (accessed on 15 October 2023)). Transmembrane helix structures were predicted using TMHMM 2.0 (https://services.healthtech.dtu.dk/services/TMHMM-2.0/ (accessed on 15 November 2023)). The presence of signal peptides was assessed using SignalP 6.0 (https://services.healthtech.dtu.dk/services/SignalP-6.0/ (accessed on 15 October 2023)). Prediction of domains and important sites of proteins was conducted using InterPro web tools (https://www.ebi.ac.uk/interpro/about/interproscan/ (accessed on 15 November 2023)). Homologous protein sequences of chitin metabolism genes from various insect species were retrieved using NCBI blastp (https://blast.ncbi.nlm.nih.gov/Blast.cgi (accessed on 15 November 2023)) for phylogenetic analysis. The neighbor-joining (NJ) method implemented in MEGA11 software [27] was used to construct phylogenetic trees with 1000 bootstrap replicates; the resulting tree files were visualized using Evolview (https://www.evolgenius.info/evolview/ (accessed on 15 December 2023)) [28]. The insect protein sequences used for phylogeny analyses are listed in Supplementary Tables S2–S6.

### 2.3. RT-PCR Analysis of Genes

Total RNA was extracted from *B. tabaci* using a TRIzol Reagent (Invitrogen, Carlsbad, CA, USA) according to the manufacturer’s instructions. A Microdrop microvolume spectrophotometer (BIO-DL, Shanghai, China) was employed to measure the absorbance ratios A260/280 and A260/230, which were used to determine the concentration and purity of RNA. The integrity of RNA was subsequently assessed using 1% agarose gel electrophoresis. A HiFiScript gDNA Removal RT MasterMix kit (CWBIO, Taizhou, China) was utilized for reverse transcription to synthesize the first-strand cDNA. Gene-specific primer pairs (see Supplementary Table S1) were designed using the Primer Premier 6.0 program. A PrimeSTAR Max DNA Polymerase (Takara, Dalian, China) was employed to amplify the coding sequences. The PCR products recovered from the agarose gel were ligated with a pTOPO-TA/Blunt vector (Aidlab, Beijing, China) for dideoxy sequencing (Sangon, Shanghai, China). After sequence alignment, the cDNA sequences were confirmed and deposited in GenBank under accession numbers PX612225–PX612229.

### 2.4. Expression Pattern Analysis of Genes

Insect individuals from various developmental stages of *B. tabaci*, including eggs, first–third instar nymphs, 1–5-day-old fourth instar nymphs, and 1–3-day-old adults, were collected for RNA extraction and reverse transcription of cDNA. For each developmental stage, approximately 100 eggs, 100 first- or second-instar nymphs, 20 third or fourth instar nymphs, or 20 adults were collected and used for total RNA extraction. The reagents used for reverse transcription and total RNA extraction were the same as those described in Section 2.3. The expression levels of chitin metabolism genes were assessed using reverse transcription quantitative PCR (RT-qPCR) with a ChamQ Blue Universal SYBR qPCR Master Mix (Vazyme, Nanjing, China) according to the manufacturer’s instructions and an FQD-96A fluorescence PCR thermocycler (Bioer, Hangzhou, China). The elongation factor 1α (EF1α) gene (GenBank accession: EE600682) was utilized as an internal reference. The primers used for RT-qPCR are listed in Supplementary Table S1. RT-qPCR was performed on each sample in triplicate, and the expression levels of target genes were calculated using the 2^−ΔΔCt^ method [29].

### 2.5. Design of Device for Insect Rearing and RNAi

A fresh cotton leaf of an appropriate size was placed flat on the surface of autoclaved 1% agar inside a sterile Petri dish (55 mm diameter, 15 mm height) once the agar cooled to room temperature. A 1-mL diet solution (composed of 5% yeast extract and 30% sucrose [wt/vol]) was applied to the outer surface of a piece of stretched Parafilm membrane (PM-996, Amcor, Neenah, WI, USA) covering one end of a glass tube (90 mm in diameter, 80 mm in height), which was then sealed with another layer of stretched Parafilm to enclose the solution between the Parafilm layers [30]. The entire setup was placed in a Petri dish (94 mm in diameter, 16 mm in height) and used for rearing fourth instar *B. tabaci* nymphs. One hundred fourth instar nymphs were collected under a stereomicroscope (Soptop, Ningbo, China) and reared in the device. The rearing experiments were performed in triplicate. The phenotypes of the whiteflies were observed every 12 h, and nymph mortality was recorded every 24 h.

### 2.6. Synthesis of dsRNA and RNA Interference

Three hundred-base pair (bp) DNA fragments were selected from the open reading frames (ORFs) of the five chitin metabolism genes to be templates for dsRNA synthesis. Specific primers with flanking T7 promoter sequences were designed (see Supplementary Table S1). dsRNA was prepared using a T7 RNAi Transcription Kit (Vazyme, Nanjing, China). The quality of the dsRNA was verified by 1% agarose gel electrophoresis and assessed using a microvolume spectrophotometer. The encoding sequence of enhanced green fluorescent protein (EGFP) was used as a template for in vitro transcription of dsRNA, which served as a negative control in the RNAi experiments.

The nanomaterial SPc was synthesized and provided by Professor Jie Shen (College of Plant Protection, China Agricultural University). Both dsRNA samples and the nanomaterial SPc were diluted in DEPC-treated water to a final concentration of 6 μg/μL. Equal volumes of the dsRNA and SPc solutions were then mixed thoroughly and incubated at 25 °C for 15 min to allow for the formation of dsRNA/SPc complexes. In the resulting complexes, the final concentrations of both dsRNA and SPc were 3 μg/μL. This solution was then diluted threefold with DEPC-treated water to prepare dsRNA/SPc (1 μg/μL). In dsRNA samples fully encapsulated by SPc; no migration of free dsRNA was observed during agarose gel electrophoresis.

In the RNAi experiments, three treatment groups were established: dsRNA (3 μg/μL), dsRNA/SPc (3 μg/μL), and dsRNA/SPc (1 μg/μL). Nymphs treated with dsEGFP (3 μg/μL) or dsEGFP/SPc (3 μg/μL) served as controls. Forty early-stage fourth instar nymphs were transferred to the insect rearing device, and 10 nanoliters of the dsRNA or dsRNA/SPc mixture was applied to the notum of the nymphs using a NANOLITER2020 nanoliter injector (WPI, Sarasota, FL, USA). The treated insects were then reared under the same conditions as described in Section 2.1. At 24, 48, and 72 h post-treatment, live *B. tabaci* individuals were collected for total RNA extraction and RT-qPCR analysis. Concurrently, additional groups of 40 nymphs were subjected to the same treatments and were observed for 5 days. The eclosion rate of *B. tabaci* adults and mortality rate of nymphs were calculated based on these observations, and the phenotypes of dsRNA-treated insects were photographed using a stereomicroscope. All treatments were performed in triplicate.

Fusion dsRNA was designed to simultaneously silence three genes: *BtNAG1*, *BtNAGK*, and *BtUAP*. These targets were selected based on their performance in gene silencing efficacy and their ability to suppress nymph and adult eclosion. Three 100-bp fragments corresponding to the three genes were tandemly concatenated in the order of BtNAG1-BtNAGK-BtUAP to form a 300-bp fusion DNA fragment (designated as *GKP*). The nucleotide sequence of *GKP* was chemically synthesized (Sangon, Shanghai, China). In vitro transcription of *GKP* dsRNA and subsequent RNAi experiments were conducted using 30 ng of dsRNA or 10 nL of the dsGKP/SPc (3 μg/μL) in parallel with its single-target counterparts dsBtNAG1/SPc (30 ng), dsBtNAGK/SPc (30 ng), and dsBtUAP/SPc (30 ng). All other aspects of the experimental setup were similar to these outlined for the single target RNAi experiments. All treatments were performed in triplicate.

### 2.7. Statistical Analysis

All experimental data were statistically analyzed using GraphPad Prism 10 (GraphPad Software Inc., San Diego, CA, USA). One-way ANOVA followed by Tukey’s test was employed to assess the expression levels of the target genes at different developmental stages. Comparisons between the various dsRNA treatment groups and the control group regarding gene expression levels and mortality rates were conducted using two-way ANOVA and Dunnett’s multiple comparisons test. For data that did not meet the assumptions of normality or homogeneity of variance, the non-parametric Kruskal–Wallis H test was applied. A *p*-value of less than 0.05 was considered statistically significant.

## 3. Results

### 3.1. Bioinformatic Analyses of Genes

**Figure 1:**
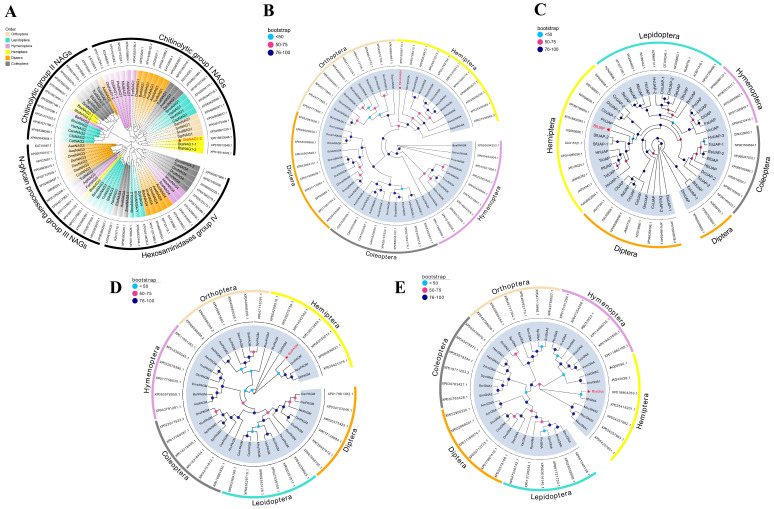
Phylogenetic Analysis of Enzymes Involved in Chitin Metabolism in *B. tabaci*. (**A**) Phylogenetic analysis of BtNAG1 and other insect NAGs. (**B**) Phylogenetic analysis of BtNAGK and other insect NAGKs. (**C**) Phylogenetic analysis of BtUAP and other insect UAPs. (**D**) Phylogenetic analysis of BtPAGM and other insect PAGMs. (**E**) Phylogenetic analysis of BtGNA and other insect GNAs. Red stars indicate proteins encoded by genes used in this study. A bootstrap analysis of 1000 replicates was applied, and bootstrap values are shown as color-shaded circles in the cladograms.

A search for *BtNAG* within the whole genome transcript data of *B. tabaci* yielded 13 sequences encoding six distinct proteins, while both *BtNAGK* and *BtUAP* each had only one transcript. To investigate the evolutionary relationships of NAGs, six putative BtNAG sequences and 69 additional NAG sequences from insects of the orders Coleoptera, Hemiptera, Hymenoptera, Diptera, Lepidoptera, and Orthoptera were used to construct a phylogenetic tree. The results were consistent with the classification of insect NAGs into four groups: chitin degradation (Group I), chitin synthesis (Group II), N-glycan processing (Group III), and hexosaminidase (Group IV) [31,32,33,34]. Among the grouped proteins, nearly all members of Groups I, II, and III were found to contain signal peptide sequences, as revealed by SignalP 6.0. BtNAG1 (GenBank number: XP018903548, corresponding to mRNA XM019048003), the only *B. tabaci* protein predicted to have a signal peptide in Group I NAGs (Table 1), was used in this study (Figure 1A).

Phylogenetic analysis of NAGK protein sequences from over 30 species across six orders showed that the putative BtNAGK was most closely related to NAGKs from *Aphis craccivora* and *Aphis gossypii* (with a bootstrap value of 97%) (Figure 1B). Phylogenetic analysis of the putative BtUAP and 45 other insect UAPs revealed that BtUAP clustered well with other Hemiptera UAPs and was most closely related to those of *Sogatella furcifera* and *Homalodisca vitripennis* (Figure 1C).

A search for *BtPAGM* within the whole genome transcript data identified three transcripts. Two of these transcripts (GenBank Accession: XM019057905 and XM019057906) share an identical ORF and contain an in-frame truncation of 300 base pairs compared to XM019054883, which was used in this study. Phylogenetic analysis of PAGM proteins from 35 insect species showed that the putative BtPAGM was located at the base of the branch where *PAGMs* from *Microplitis demolitor*, *Neodiprion virginianus*, and *Hylaeus volcanicus* were located (with a bootstrap value of 99%), indicating that *BtPAGM* diverged the earliest from the common ancestor of this lineage (Figure 1D). In addition, MEME analysis revealed 10 highly conserved protein motifs in the PAGM protein of *B. tabaci*, which were also widely present in the PAGM homologs of the other 34 insect species (Supplementary Figure S1).

A search for *BtGNA* within the whole-genome transcript data yielded two transcripts. Sequence alignment of the ORFs of the two BtGNAs (GenBank Accession: XM019048718 and XM019048719) revealed that XM019048718 is a truncated version of XM019048719, lacking a 36-bp 5′ fragment. The latter was selected for RNAi targeting. Phylogenetic analysis of GNA sequences from 33 insect species revealed that BtGNA forms an evolutionary branch with other two hemipteran GNAs: SfGNA from *Sogatella furcifera* and ApGNA from *Acyrthosiphon pisum* (Figure 1E).

The gene names, their GenBank accession numbers, subcellular localization, the protein domain families, and other predicted feature are listed in Table 1.

### 3.2. Temporal Gene Expression Profiles

**Figure 2:**
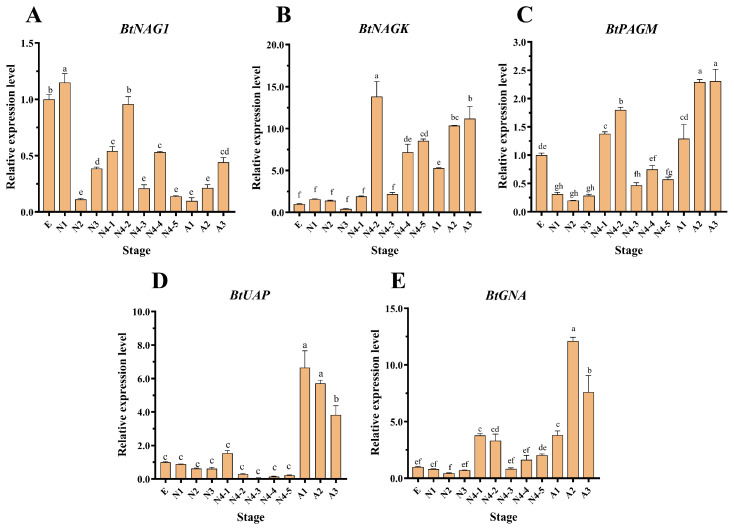
(**A**–**E**) Expression of *BtNAG1*, *BtNAGK*, *BtPAGM*, *BtUAP*, and *BtGNA* at various developmental stages in *B. tabaci*. E, Egg; N1–3, 1st–3rd instar nymph; N4-1–N4-4, 1st–4th day fourth instar nymph; A1–A3, 1st–3rd day adults. Each bar represents the mean ± SD. Different letters above the bars indicate significant differences at *p* < 0.05 based on Duncan’s test.

The expression levels of *BtNAG1*, *BtNAGK*, *BtPAGM*, *BtUAP*, and *BtGNA* in different developmental stages of *B. tabaci* were determined through RT-qPCR. *BtNAG1* exhibited highest expression levels during the egg and the first and fourth instar nymph stages (Figure 2A), whereas *BtNAGK* and *BtPAGM* were mainly expressed in the fourth instar nymph and adult stages (Figure 2B,C). Additionally, *BtUAP* and *BtGNA* showed predominant expression in the adult stage (Figure 2D,E).

### 3.3. Validation of Insect Rearing and RNAi Device

**Figure 3:**
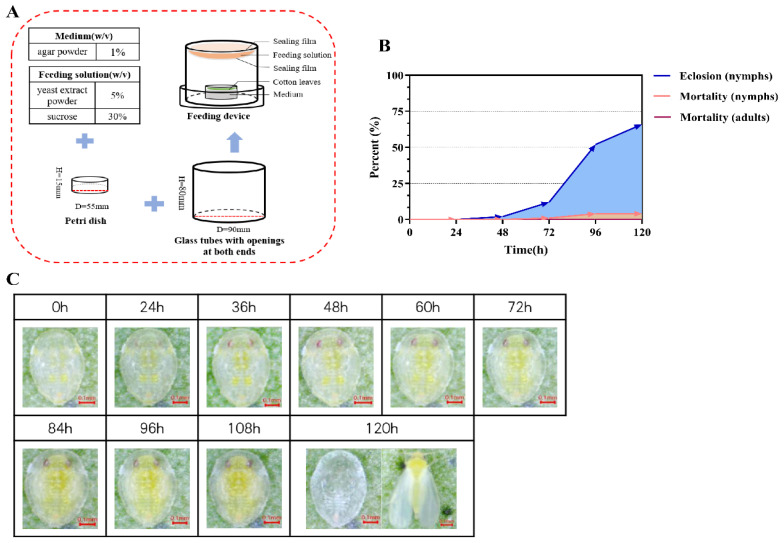
Validation of the whitefly rearing and RNAi device. (**A**) Components of the device. (**B**) Mortality and emergence rates of fourth instar nymphs after feeding, and mortality rates after emergence of nymphs. (**C**) Photos of the fourth instar whitefly nymphs during feeding. Photos of 0 to 108 h, recording sequential changes in 4th-instar nymphs, whose body color changed from light yellow to yellow-green, eyes enlarged with a deeper red hue, and wings became visible in the late 4th-instar stage. Photos at 120 h of a nymphal exuviae (left) and an emerged adult whitefly (right).

Using the previously described device (Figure 3A), we reared and observed 100 fourth instar whitefly nymphs for 120 h and recorded the normal developmental transitions prior to the eclosion of adults with a camera. During this process, the nymphs changed their body color from light yellow to yellow-green. Their eyes enlarged and took on a deeper red hue, and the wings became visible in the late fourth instar stage (Figure 3C). The duration of the fourth instar stage was approximately five days. During the first 48 h, the adult eclosion rate of fourth instar nymphs was near zero, followed by a marked increase. During the 72 to 96 h period, the eclosion rate reached 40%, and after 120 h, it rose to approximately 66% (Figure 3B). The mortality rate of the nymphs remained relatively low throughout the observation period. Overall, the majority of fourth instar nymphs in the rearing device successfully eclosed, with a survival rate of up to 95% at 120 h (Figure 3B).

### 3.4. Expression of Target Genes After dsRNA Treatment

**Figure 4:**
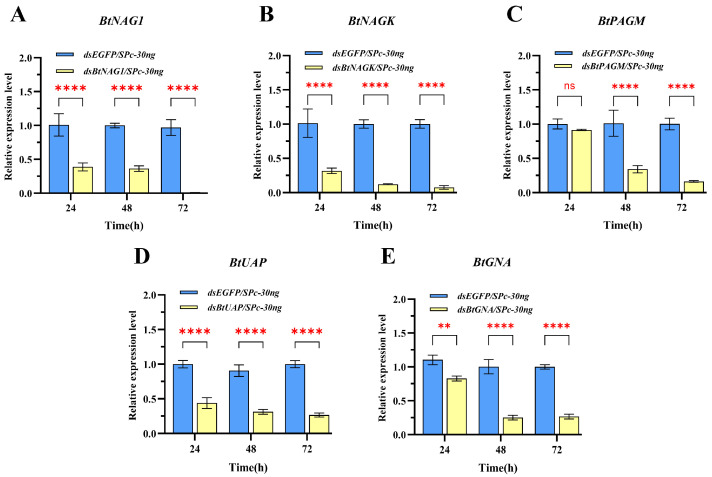
Expression of target genes at 24 h, 48 h, and 72 h after treatment with SPc-mediated dsRNA. (**A**) Expression of *BtNAG1* after treatment with dsBtNAG1/SPc complex. (**B**) Expression of *BtNAGK* after treatment with dsBtNAGK/SPc complex. (**C**) Expression of *BtPAGM* after treatment with dsBtPAGM/SPc complex. (**D**) Expression of *BtUAP* after treatment with dsBtUAP/SPc complex. (**E**) Expression of *BtGNA* after treatment with dsBtGNA/SPc complex. 30 ng of dsRNA and 30 ng of SPc were applied per nymph. The mass ratio of SPc to dsRNA was 1:1. Error bars represent the standard error of the mean (n = 3). Each bar represents the mean ± SD. Significance levels were determined using two-way ANOVA with Dunnett’s multiple comparisons; asterisks indicate statistically significant differences (** *p* < 0.01, **** *p* < 0.0001). “ns” stands for no significant difference.

**Figure 5:**
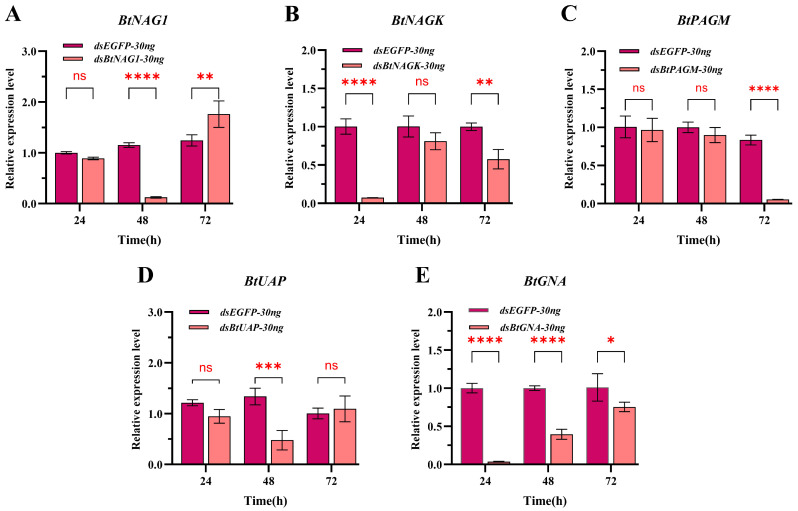
Expression of target genes at 24 h, 48 h, and 72 h after treatment with naked dsRNA. (**A**) Expression of *BtNAG1* after treatment with dsBtNAG1. (**B**) Expression of *BtNAGK* after treatment with dsBtNAGK. (**C**) Expression of *BtPAGM* after treatment with dsBtPAGM. (**D**) Expression of *BtUAP* after treatment with dsBtUAP. (**E**) Expression of *BtGNA* after treatment with dsBtGNA. Error bars represent the standard error of the mean (n = 3). 30 ng of dsRNA was used per whitefly nymph. Each bar represents the mean ± SD. Significance was determined using two-way ANOVA with Dunnett’s multiple comparisons; asterisks indicate statistically significant differences (* *p* < 0.05, ** *p* < 0.01, *** *p* < 0.001, **** *p* < 0.0001). “ns” stands for no significant difference.

In the SPc-mediated dsRNA-treated groups, except for the dsBtPAGM/SPc-30 ng group at 24 h, the expression levels of the four other groups at the three sampling time points were significantly lower than these of the control group treated with dsEGFP/SPc-30 ng and the silencing of genes persisted for over 48 h post-treatment (Figure 4). From 24 to 72 h post-treatment, the expression levels of *BtNAG1*, *BtNAGK*, *BtPAGM*, *BtUAP*, and *BtGNA* were suppressed by approximately 63.64% to 99.16%, 68.61% to 92.54%, 8.76% to 83.81%, 56.12% to 73.01%, and 17.11% to 73.30%, respectively (Figure 4). In the naked dsRNA-treated groups (Figure 5), suppression of target gene expression was first detected at 24 h (*BtNAGK* and *BtGNA*), 48 h (*BtNAG1* and *BtUAP*), and 72 h (*BtPAGM*). However, the suppression was transient and expression levels of the target genes mostly recovered within 24 h (*BtNAG1*, *BtNAGK*, *BtUAP*, and *BtGNA*). Overall, an earlier onset and more persistence of gene silencing were achieved using the nanocarrier-delivered gene silencing (NDGS) system compared to using naked dsRNA, highlighting the capacity of SPc nanomaterials to enhance RNAi efficacy.

### 3.5. Mortality and Adult Emergence Rates of Whitefly Nymphs After RNAi

**Figure 6:**
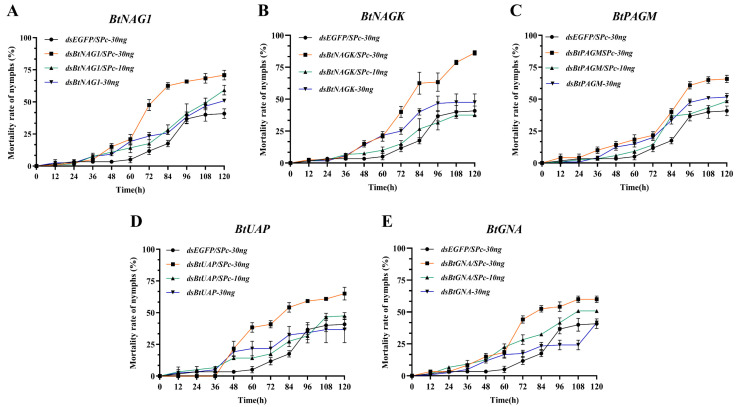
Mortality rates of fourth instar *B. tabaci* nymph after dsRNA/Spc treatments. (**A**) Mortality rates of nymph after treatment with dsBtNAG1/SPc. (**B**) Mortality rates of nymph after treatment with dsBtNAGK/SPc. (**C**) Mortality rates of fourth instar nymph after treatment with dsBtPAGM/SPc. (**D**) Mortality rates of fourth instar nymph after treatment with dsBtUAP/SPc. (**E**) Mortality rates of fourth instar nymphs after treatment with dsBtGNA/SPc. Each treatment consisted of 40 fourth instar *B. tabaci* nymphs, with mortality rates recorded every 12 h over a period of 5 days. Statistical significance was assessed using two-way ANOVA followed by Dunnett’s multiple comparison test.

Within the first 24 h after dsRNA treatment, the mortality rates of nymphs in all groups were close to 0 (Figure 6A–E). In most groups, the mortality rate of nymphs remained relatively low and increased gradually before 36 h. At 120 h, the nymph mortality rates in the groups treated with dsRNA/SPc-30 ng against the target genes reached levels ranging from 58% to 86.25% (Figure 6B,E), which were significantly higher than these in most of other groups at 60 h post-interference. Meanwhile, compared to the naked dsRNA groups, the SPc-mediated groups showed an increase in nymph mortality rates ranging from 14.2% to 38.75% at 120 h. Among them, the groups targeting *BtNAGK*, *BtUAP*, and *BtNAG1* showed the strongest effects, with increases in mortality rates of 38.75%, 25.9%, and 20%, respectively (Figure 6A,B,D).

**Figure 7:**
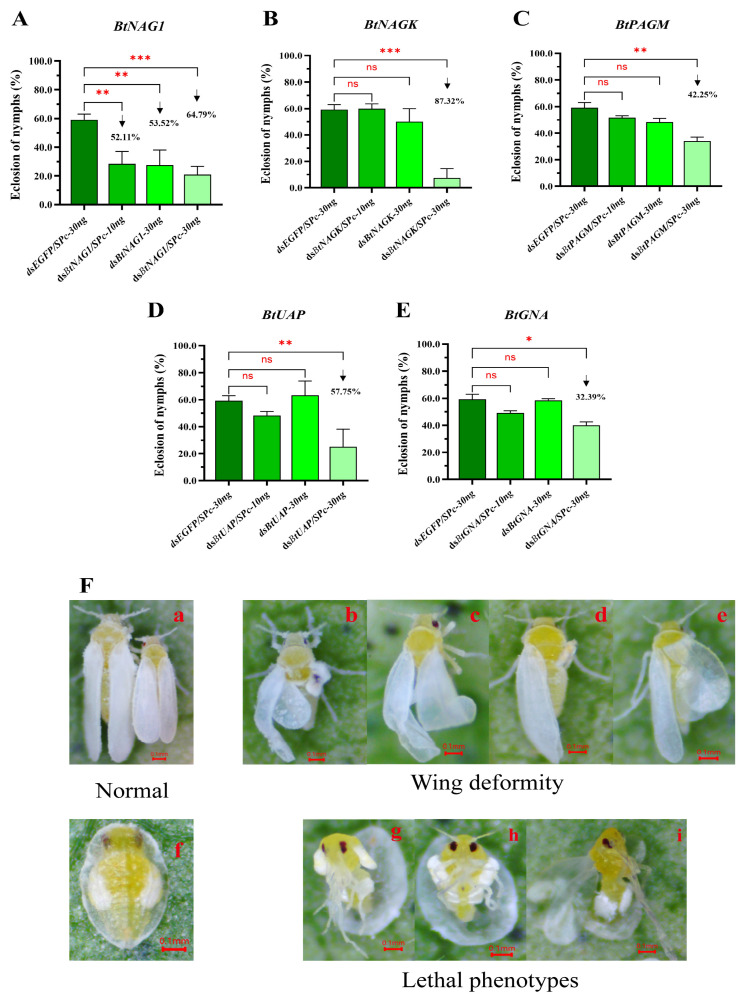
Adult emergence rates and phenotypes of fourth instar *B. tabaci* nymph after dsRNA treatments. (**A**–**E**) Statistical analysis of the adult emergence rates of fourth instar nymph after treatment with dsBtNAG1, dsBtNAGK, dsBtPAGM, dsBtUAP, and dsBtGNA. (**F**) Insect phenotypes after treatment with dsBtNAG1/SPc-30ng. (**F**(**a**),**F**(**f**)): Normal phenotypes of adults and fourth instar nymphs; (**F**(**b**)–**F**(**e**)): Nymphs able to emerge as adults but with curled wings that failed to expand fully; (**F**(**g**)–**F**(**i**)): nymphs with old cuticle ruptured but they are unable to fully molt, leading to death. Each bar represents the mean ± SD. The significance levels for *BtGNA* and *BtPAGM* were assessed using the Kruskal–Wallis non-parametric test, while the significance levels for *BtNAG1*, *BtNAGK*, and *BtUAP* were determined using one-way ANOVA and Dunnett’s multiple comparison test. Asterisks indicate statistically significant differences (* *p* < 0.05, ** *p* < 0.01, *** *p* < 0.001). “ns” stands for no significant difference. The downward arrow indicates a decline.

Silencing target genes for 120 h using dsRNAs, with or without SPc mediation, affected the adult emergence rates of fourth instar *B*. *tabaci* nymphs (Figure 7A–E). However, most of groups treated with 10 ng of dsRNA, whether SPc-mediated or non-SPc-mediated, did not lead to a significant change in adult emergence rates within 120 h, except for the dsBtNAG1 group (Figure 7A–E). Among all the groups, the dsRNA/SPc-30ng treatment group showed the strongest inhibitory effects on adult emergence rates (Figure 7A–E). In all 120-h dsBtNAG1 treatment groups of dsBtNAG1, dsBtNAG1/SPc-10ng, dsBtNAG1-30ng, and dsBtNAG1/SPc-30ng, significant changes in adult emergence were observed. Overall, complexing 30 ng of dsRNAs to SPc, including dsBtNAGK, dsBtPAGM, dsBtUAPl, and dsBtGNA, greatly enhanced the inhibitory effects on the emergence rate of whitefly adults.

Compared to the control group, the adult emergence rates in the dsRNA/SPc 30 ng treatment groups were significantly lower, and there was a higher deformity rate observed in the whiteflies, particularly in the dsBtNAG1, dsBtNAGK, and dsBtUAP treatment groups. Whitefly individuals with phenotypic deformities were mostly observed in the BtNAG1/SPc-30ng (9/120) (Figure 7F) and BtNAGK/SPc-30ng (8/120) groups. Two types of phenotypic deformity were observed: young adult individuals with curled wings that failed to fully expand (Figure 7F(b)–F(e)) and nymphs with ruptured old cuticle but they failed to molt, leading to death (Figure 7F(g)–F(i)).

### 3.6. Fusion Gene RNAi Efficiency

**Figure 8:**
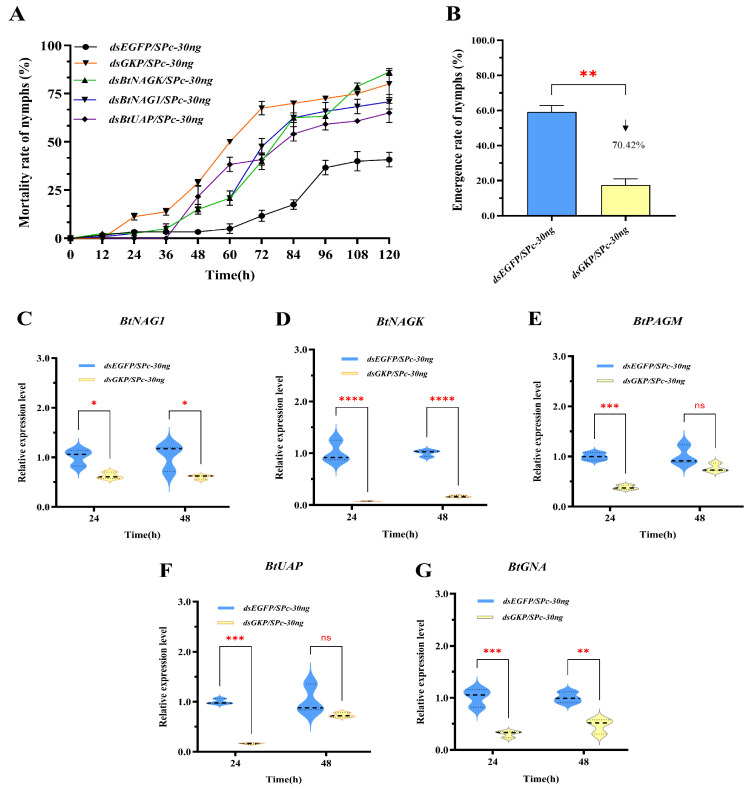
Nymph mortality rate, adult emergence rate, and expression levels of target genes in fourth instar *B. tabaci* nymphs following treatment with dsRNA targeting the fusion gene *GKP*. (**A**) Mortality rate of fourth instar nymphs after dsGKP treatment. (**B**) Adult emergence rate of fourth instar nymphs after dsGKP treatment. (**C**–**G**) Expression of *BtNAG1*, *BtNAGK*, *BtPAGM*, *BtUAP*, and *BtGNA* at 24 and 48 h post dsGKP treatment. The significance of changes in mortality rates and gene expression levels was assessed using a two-way ANOVA, while emergence rates were analyzed with a one-way ANOVA. All analyses were followed by Dunnett’s multiple comparison test. Asterisks indicate statistically significant differences (* *p* < 0.05, ** *p* < 0.01, *** *p* < 0.001, **** *p* < 0.0001). “ns” stands for no significant difference. The downward arrow indicates a decline.

The fusion gene *GKP* was designed and constructed using partial sequences from *BtNAG1*, *BtNAGK*, and *BtUAP* (see Supplementary Table S7). RNAi experiments were conducted using dsGKP/SPc-30ng, along with its single-target version (dsBtNAG1/SPc-30ng, dsBtNAGK/SPc-30ng, and dsBtUAP/SPc-30ng), on fourth instar whitefly nymphs (Figure 8). Compared with the control group, the dsGKP group showed a significant increase in nymph mortality as early as 24 h post-treatment, whereas the single-target dsRNA groups first exhibited significant increases at 48 h. The most obvious increase in nymph mortality for the single-target groups occurred between 36 and 84 h post-treatment, while for the dsGKP group, it occurred between 12 and 72 h post-treatment (Figure 8A). These results indicate that the onset of nymph mortality occurred earlier in the fusion dsRNA group than in the single-gene dsRNA groups. Furthermore, dsRNA targeting the fusion gene produced greater lethality than single-gene dsRNA in all cases except the dsBtNAGK group (Figure 8A).

The gene silencing effects of dsGKP were evaluated by measuring transcript levels using RT-qPCR at 24 and 48 h following dsRNA application. After 24 h, the expression levels of *BtNAG1*, *BtNAGK*, *BtUAP*, *BtPAGM*, and *BtGNA* decreased by 37.79%, 92.72%, 83.79%, 62.00%, and 69.69%, respectively. At 48 h, expression levels of *BtNAG1*, *BtNAGK*, *BtUAP*, *BtPAGM*, and *BtGNA* decreased by 41.17%, 83.31%, 28.60%, 23.70%, and 53.57%, respectively. The greatest reduction in gene expression was observed in *BtNAGK* at 24 h, with an expression level of 7.28% of the control (Figure 8D), followed by *BtUAP* (16.21% of control) and *BtNAG1* (62.21% of control) (Figure 8E,F). At 48 h, *BtNAGK* maintained the highest level of gene expression suppression, with an expression level 16.69% of that of the control (Figure 8D). Notably, following dsGKP treatment, significant downregulation of two non-target genes, *BtPAGM* and *BtGNA*, was detected at 24 h post-treatment, suggesting a coordinated regulation of gene expression in the chitin metabolic pathway. Similarly, RNA interference targeting two *BtTPS*s in *B. tabaci* altered the expression of several genes involved in chitin biosynthesis and energy metabolism pathways [35].

## 4. Discussion

The global invasiveness of *B. tabaci* has made it one of the most dangerous threats to world crop and vegetable production. Recent findings of horizontal gene transfers from plants and bacteria to *B. tabaci* may exemplify a crucial aspect of adaptive evolution that enhances its invasiveness [36,37]. RNAi has been utilized for the specific silencing of insect genes for nearly two decades. Its straightforward target gene selection process and high efficiency in gene silencing have greatly inspired people’s enthusiasm for the development of RNAi-based pest-control methods [38,39]. Theoretically, all genes essential for insect survival, development, growth, or defense can serve as potential RNAi targets. Therefore, RNAi-based strategy broadens the scope for screening pest control targets and accelerating the development of new control measures. However, the efficacy of RNAi can vary significantly due to several factors, such as the orders of pest insects, the specific target genes selected, the mode of dsRNA delivery, and the presence of dsRNA nucleases in the midgut [38,40,41].

RNAi targeting genes in the chitin metabolism pathway has been extensively explored and proven effective in gene silencing in various insect species [42,43]. Specifically, RNAi targeting key enzymes at different stages of this pathway—from synthesis to degradation—usually leads to lethal effects. For instance, silencing genes for trehalase (*SeTRE1/2* in *Spodoptera exigua*) or hexokinase (*DcHK* in *Diaphorina citri*) led to significant mortality and developmental deformities [44,45]. Targeting enzymes further downstream, such as GFAT, UAP, and CHS, yielded similarly significant results. Knockdown of *LmGFAT* in *Locusta migratoria* caused 70% mortality [46], while silencing *NlUAP* in *Nilaparvata lugens* led to 100% mortality [47]. The two chitin synthase isoforms also are useful RNAi targets. Silencing *TcCHS1* (cuticular) in *Tribolium castaneum* disrupted molting, whereas silencing *TcCHS2* (midgut) impaired feeding and growth [48]. RNAi targeting the chitin degradation pathway is also a viable strategy. Silencing key chitinase genes (*BtCht*) in *B*. *tabaci* increased nymphal mortality [23]. Similarly, knocking down genes for NAG and NAGK, which work with chitinases, resulted in survival rates below 17% in *Holotrichia parallela* larvae [49]. However, a notable exception is the *GNA* gene in *L. migratoria*, where RNAi-mediated silencing, despite reducing transcript levels, failed to affect molting or development [50]. This highlights the importance of target validation when developing effective RNAi-based pest-control strategies.

One limitation of these RNAi studies is that dsRNA was primarily delivered via microinjection, a method that is impractical for field applications. In our study, to mimic field spray conditions at least partially, a nanoliter injection system was used to drop dsRNA/SPc solution onto the nymphal notum of *B. tabaci*. Consequently, significant gene silencing, mortality, and developmental deformity were observed in *B. Tabaci* nymphs in our study. Our results demonstrate the efficacy of RNAi in targeting chitin-metabolic-pathway genes in *B. tabaci*.

Fusion dsRNA enables the simultaneous downregulation of multiple targets which may be difficult to achieve with conventional chemical pesticides. Furthermore, a fusion dsRNA strategy may represent an effective approach to mitigating the risk of pest resistance development caused by single-gene mutations [3,51]. Finally, the presence of two or more homologous genes with overlapping functions in eukaryotic genomes often leads to gene redundancy, which can mask loss-of-function phenotypes in RNAi and genome editing experiments [52]. Therefore, for homologous genes with low sequence similarity, RNA interference with fusion dsRNA may help overcome the gene compensation effects arising from the gene redundancy. To evaluate the fusion dsRNA strategy, we designed the GKP construct that includes segments from *BtNAG1*, *BtNAGK*, and *BtUAP*. SPc-mediated RNAi with dsGKP significantly increased nymph mortality to approximately 80% and reduced adult emergence by 70%. Our data show that dsGKP treatment elicited a stronger RNAi response than the corresponding single-target treatments up to 96 h post-treatment. However, between 96 h and 120 h, nymphal mortality in the dsNAGK group exceeded that observed in the dsGKP group (Figure 8). This may be related to the design of dsRNAs. The single-target dsRNAs were 300 bp in length, whereas the fused dsRNA was also 300 bp in total, comprising three fragments of 100 bp each. In previous studies, it has been reported that moderately increasing dsRNA length can enhance RNAi efficiency [53]. For example, in third-instar larvae of the Colorado potato beetle (*Leptinotarsa decemlineata*), foliar application of 266- or 297-bp dsRNAs targeting *actin* resulted in a greater than 90% mortality rate, whereas 50- or 102-bp dsRNAs exhibited no lethal effects on the larvae [54]. Therefore, careful optimization of dsRNA length is required when designing RNAi experiments in *T. bamisia*.

RNAi-based pest-control strategy is a new biotechnological tool. One practical form of this strategy is spray-induced gene silencing (SIGS). To enhance delivery efficiency, dsRNA can be supplemented with nanomaterials such as chitosan, SPc, CQD, and guanidylated polymers [20]. Among these, the efficacy of SPc for transdermal dsRNA delivery has been reported in a variety of insect pests, including *Grapholita molesta* [55], *Adelphocoris suturalis* [56], *Thrips palmi* [21] and *Aphis glycines* [57]. Among all the dsRNAs tested in our study, the SPc-mediated RNAi treatments produced earlier onset, more prolonged gene silencing and greater suppression of adult eclosion. SPc consists of a hydrophobic core and a positively charged hydrophilic shell that are used to encapsulate dsRNA [58]. The structure renders it capable of protecting dsRNA from degradation by RNases and facilitating intracellular delivery [59,60], as demonstrated by our RNAi results. Additionally, the cost for the large-scale production of SPc is expected to be cheap enough for field application [61]. Future field spray trials are required to confirm its practical feasibility under realistic conditions. Furthermore, the advantages and compatibility of this approach with beneficial insects, including pollinators and natural enemies of *B. Tabaci*, should be evaluated to ensure its integration into the framework of integrated pest management (IPM) [38,62].

Host-Induced Gene Silencing (HIGS) is another dsRNA delivery approach that uses engineered plants to express hairpin RNA (hpRNA) targeting essential pest genes. When feeding on these plants, the ingested hpRNA is processed by the RNAi machinery of pests into small interfering RNAs (siRNAs). These siRNAs in turn trigger sequence-specific RNAi responses in the insect pests [18,19,63]. Although generating hpRNA-expressing plants is labor-intensive and time-consuming, HIGS offers a practical and cost-efficient means of delivering dsRNA for field applications [18,19,38,63]. Furthermore, HIGS minimizes the degradation of dsRNA because hpRNA is produced in planta rather than being applied as a spray. Beyond public concern about potential environmental risks, transgenic plants expressing hpRNA targeting chitin metabolism genes remain a promising option for future trials.

## 5. Conclusions

In summary, five genes involved in chitin metabolism were identified in the *B. tabaci* genome, and their phylogenetic relationships with homologs from other insect taxa were analyzed. As revealed by RT-qPCR assays, *BtNAGK* and *BtPAGM* were mainly expressed in the fourth instar nymph and adult stages, whereas *BtUAP* and *BtGNA* were predominantly expressed in the adult stage. Additionally, *BtNAG1* exhibited the highest expression levels during the egg and the first and fourth instar nymph stages. Compared to naked dsRNA treatments, SPc-mediated RNAi targeting of these genes in fourth instar *B. tabaci* nymphs resulted in stronger and more persistent suppression of gene expression, greater suppression of adult emergence, and significant developmental deformities. Finally, RNAi using a fusion dsRNA targeting *BtNAG1*, *BtNAGK*, and *BtUAP* achieved a more rapid onset of the RNAi response. These findings demonstrated the efficacy of nanomaterial-assisted RNAi targeting chitin-metabolic-pathway genes in *B. tabaci*, providing useful insights into the development of novel pest management strategies.

## Figures and Tables

**Table 1:** Predicted features of Proteins Encoded by Candidates target genes in *B. tabaci*.

Gene Name	GenBank Accession	Aa	pI	Subcellular Localization	SP	TMHs	Protein Domain Family
*BtNAG1*	XM019048003	622	5.62	lysosome	+	-	GH20
*BtNAGK*	XM019053168	344	5.20	cytoplasm	-	-	ASKHA superfamily
*BtPAGM*	XM019054883	567	6.29	cytoplasm	-	-	α-D-phosphohexomutase superfamily
*BtUAP*	XM019046508	492	6.30	cytoplasm	-	-	GT superfamily-A
*BtGNA*	XM019048719	173	7.62	cytoplasm	-	-	N-Acyltransferase superfamily

Note: SP indicates a signal peptide; TMHs indicate transmembrane helices; + and - indicate presence and absence.

## Data Availability

All data in this study, such as the gene entry numbers, are available on the NCBI (National Center for Biotechnology Information) website.

## Supplementary Materials

The following supporting information can be downloaded at: https://www.mdpi.com/article/10.3390/insects17010002/s1, Table S1: Primers used in this study; Table S2: Information on insect NAG proteins; Table S3: Information on insect NAGK proteins; Table S4: Information on insect UAP proteins; Table S5: Information on insect PAGM proteins; Table S6: Information on insect GNA proteins; Table S7: Design of 300-bp dsGKP template; Figure S1: Analysis of conserved motifs in insect PAGM proteins.
