# Supplementary material for: Nanomaterial-Mediated RNAi Targeting Chitin Metabolism Genes in MEAM1 Cryptic Species of Bemisia tabaci (Hemiptera: Aleyrodidae)
Source: Insects. 2025 Dec 19;17(1):2. doi: 10.3390/insects17010002 (PMC12842457; doi:10.3390/insects17010002)
Supplement: Supplementary file 1 [file insects-17-00002-s001.zip › insects-4023553-supplementary.pdf]

Table S1. Primers used in this study.

PCR reactions	Primer name	Primer sequence (5'-3')
PCR	BtNAG1-F	ATGGCGATTAGGTGGAAGAGTC
	BtNAG1-R	CTAGGTGGTCAGGTAACAACTTCC
	BtNAGK-F	AATTGCTTGAGGAAGCTCTTGAAC
	BtNAGK-R	GCTTGCTACCTGGGCTAAGC
	BtPAGM-F	GAACTGGTCCCTGCCTCTAC
	BtPAGM-R	AGAATGCTAAAGAGGGTGTTCA
	BtUAP-F	GTTGTCAGTGTTGTGCGAAAGG
	BtUAP-R	AAATACTTAGTGCTGGCAGTTTGG
	BtGNAT-F	TTCTGCAACCAAGTGTAGGATCG
	BtGNAT-R	TGAACCATTCTGAGAGTCACAGTC
RT-qPCR	BtNAG1-F	CCGATGTCGTCAGTATCAAC
	BtNAG1-R	TTATGCCCAGGGTCTTTCAG
	BtNAGK-F	AATTAACTGTCAGCGTGTCG
	BtNAGK-R	GGGCGTTTATAGTGGAAGAAG
	BtPAGM-F	GACAAGTCACCGAAGTATAGC
	BtPAGM-R	GGTAAGCAACATGGCCTAAA
	BtUAP-F	GCATTACAAGACAACGGACTC
	BtUAP-R	CAAGAGCTTCAGTAGGAGATG
	BtGNAT-F	GAATGTGGAATACGAGGTCG
	BtGNAT-R	CGGCACTAGGCTATCTTTAC
	BtEF1α-F	GTAATGCTGAAGGTAAATG
	BtEF1α-R	CAAAAGTGACAACCATAC
dsRNA synthesis	BtNAG1-F	TAATACGACTCACTATAGGGCTACGAGAACGACCCGATGA
	BtNAG1-R	TAATACGACTCACTATAGGGCAACTTCCCTCATTCTGGTAGC
	BtNAGK-F	TAATACGACTCACTATAGGGATCACTCTTCAATGGCAAAGGT
	BtNAGK-R	TAATACGACTCACTATAGGGTTATGAGCAACAGCGATTGGC
	BtPAGM-F	TAATACGACTCACTATAGGGTGTCCTCAACAACGAATGCG
	BtPAGM-R	TAATACGACTCACTATAGGGTTTGTGGAGCTGCCATTTGC
	BtUAP-F	TAATACGACTCACTATAGGGCATTACAAGACAACGGACTCCTGAG
	BtUAP-R	TAATACGACTCACTATAGGGAAATGCTCCCTGCCCTGAAAGT
	BtGNAT-F	TAATACGACTCACTATAGGGGGTGATGTTACGTTTGATCAGTTC
	BtGNAT-R	TAATACGACTCACTATAGGGCACTAGGCTATCTTT

	EGFP-F	ACACTCCAG<br>TAATACGACTCACTATAGGGCTGGAGTTGTCCCA<br>ATTCTTGTTGA

Table S2. Information of insect NAG proteins.

Accession number	Species	Gene Name	Order
RZC37715.1	*Asbolus verrucosus*	AveNAG4	Coleoptera
RZC43025.1	*Asbolus verrucosus*	AveNAG1	Coleoptera
XP048521464.1	*Dendroctonus ponderosae*	DpoNAG1	Coleoptera
XP057662279.1	*Diorhabda carinulata*	DcaNAG4	Coleoptera
XP057662766.1	*Diorhabda carinulata*	DcaNAG2	Coleoptera
XP056632229.1	*Diorhabda sublineata*	DsuNAG4	Coleoptera
XP066150143.1	*Euwallacea fornicatus*	EfoNAG2	Coleoptera
XP066150433.1	*Euwallacea fornicatus*	EfoNAG4	Coleoptera
XP050293579.1	*Anthonomus grandis grandis*	AgrNAG2	Coleoptera
XP001092296.1	*Tribolium castaneum*	TcaNAG4	Coleoptera
XP001092298.1	*Tribolium castaneum*	TcaNAG3	Coleoptera
XP001092318.1	*Tribolium castaneum*	TcaNAG1	Coleoptera
XP975656.1	*Tribolium castaneum*	TcaNGA2-1	Coleoptera
XP975660.1	*Tribolium castaneum*	TcaNGA2-2	Coleoptera
XP028129173.1	*Diabrotica virgifera virgifera*	DviNAG4	Coleoptera
XP063909245.1	*Zophobas morio*	ZmoNAG4-1	Coleoptera
XP063921966.1	*Zophobas morio*	ZmoNAG4-2	Coleoptera
EAT36388.1	*Aedes aegypti*	AaeNAG4	Diptera
EAT40440.1	*Aedes aegypti*	AaeNAG3	Diptera
EAT43655.1	*Aedes aegypti*	AaeNAG2	Diptera
EAT43909.1	*Aedes aegypti*	AaeNAG1	Diptera
XP050100152.1	*Anopheles aquasalis*	AaqNAG3	Diptera
XP049463823.1	*Anopheles coluzzii*	AcoNAG4	Diptera
XP058124962.1	*Anopheles coustani*	AcoNAG1	Diptera
XP011207588.2	*Bactrocera dorsalis*	BdoNAG4	Diptera
XP014099182.2	*Bactrocera oleae*	BolNAG1	Diptera
JAB96203.1	*Ceratitis capitata*	CcaNAG3	Diptera
JAC00207.1	*Ceratitis capitata*	CcaNAG1	Diptera
XP026838315.1	*Drosophila erecta*	DerNAG3	Diptera
XP523924.1	*Drosophila melanogaster*	DmeNAG1	Diptera
XP525081.1	*Drosophila melanogaster*	DmeNAG3	Diptera
XP725178.1	*Drosophila melanogaster*	DmeNAG4	Diptera
XP060658402.1	*Drosophila nasuta*	DnaNAG4	Diptera
XP020808497.1	*Drosophila serrata*	DseNAG3	Diptera
XP037727505.1	*Drosophila subpulchrella*	DsuNAG3	Diptera
XP058830827.1	*Topomyia yanbarensis*	TyaNAG1	Diptera
XP018903548.1	*Bemisia tabaci*	BtaNAG1-2	Hemiptera
XP018909837.1	*Bemisia tabaci*	BtaNAG2-1	Hemiptera

XP018911129.1	*Bemisia tabaci*	BtaNAG3	Hemiptera
XP018913348.1	*Bemisia tabaci*	BtaNAG1-1	Hemiptera
XP018914551.1	*Bemisia tabaci*	BtaNAG2-2	Hemiptera
XP018914878.1	*Bemisia tabaci*	BtaNAG1-3	Hemiptera
XP044017062.1	*Aphidius gifuensis*	AgiNAG3	Hymenoptera
XP061935597.1	*Apis cerana*	AceNAG4	Hymenoptera
XP043789904.1	*Apis laboriosa*	AlaNAG1	Hymenoptera
XP050587198.1	*Bombus affinis*	BafNAG2	Hymenoptera
XP011347841.3	*Cerapachys biroi*	ObiNAG1-2	Hymenoptera
XP011315001.1	*Fopius arisanus*	FarNAG3	Hymenoptera
XP043520160.1	*Frieseomelitta varia*	FvaNAG1	Hymenoptera
XP014296336.1	*Microplitis demolitor*	MdeNAG4	Hymenoptera
XP057328369.1	*Microplitis mediator*	MmeNAG4	Hymenoptera
XP029175324.1	*Nylanderia fulva*	NfuNAG1	Hymenoptera
EZA48639.1	*Ooceraea biroi*	ObiNAG1-1	Hymenoptera
XP011635262.1	*Pogonomyrmex barbatus*	PbaNAG1	Hymenoptera
XP023946294.1	*Bicyclus anynana*	BanNAG3	Lepidoptera
XP001037096.1	*Bombyx mori*	BmoNAG2	Lepidoptera
XP001037466.1	*Bombyx mori*	BmoNAG1	Lepidoptera
XP001078833.1	*Bombyx mori*	BmoNAG3	Lepidoptera
XP001165928.1	*Bombyx mori*	BmoNAG4-2	Lepidoptera
XP004922444.1	*Bombyx mori*	BmoNAG4-1	Lepidoptera
XP063371186.1	*Cydia amplana*	CamNAG2	Lepidoptera
XP063386046.1	*Cydia fagiglandana*	CfaNAG2	Lepidoptera
XP063625588.1	*Cydia splendana*	CspNAG1	Lepidoptera
XP063540854.1	*Cydia strobilella*	CstNAG2	Lepidoptera
XP021201532.2	*Helicoverpa armigera*	HarNAG2	Lepidoptera
XP063892907.1	*Helicoverpa armigera*	HarNAG3	Lepidoptera
XP050347018.1	*Nymphalis io*	NioNAG1	Lepidoptera
XP032519636.2	*plexippus plexippus*	DplNAG4	Lepidoptera
XP022830074.1	*Spodoptera litura*	SliNAG3	Lepidoptera
XP026737934.1	*Trichoplusia ni*	TniNAG3	Lepidoptera
XP026484110.1	*Vanessa tameamea*	VtaNAG1	Lepidoptera
XP046992971.1	*Schistocerca americana*	SamNAG1	Orthoptera
XP049778356.1	*Schistocerca cancellata*	ScaNAG1	Orthoptera
XP049861030.1	*Schistocerca gregaria*	SgrNAG1	Orthoptera
XP049808657.1	*Schistocerca nitens*	SniNAG1	Orthoptera

Table S3. Information of insect NAGK proteins.

Accession number	Species	Gene Name	Order
CAK1620095.1	*Acanthoscelides obtectus*	AcobtNAGK-1	Coleoptera
CAK1620097.1	*Acanthoscelides obtectus*	AcobtNAGK-2	Coleoptera
CAK1620096.1	*Acanthoscelides obtectus*	AcobtNAGK-3	Coleoptera
CAK1620098.1	*Acanthoscelides obtectus*	AcobtNAGK-4	Coleoptera

XP028131932.1	*Diabrotica virgifera*	DivirNAGK	Coleoptera
XP057652178.1	*Diorhabda carinulata*	DicarNAGK	Coleoptera
XP056635192.1	*Diorhabda sublineata*	DisubNAGK	Coleoptera
XP023029017.1	*Leptinotarsa decemlineata*	LedecNAGK-1	Coleoptera
XP023029015.1	*Leptinotarsa decemlineata*	LedecNAGK-2	Coleoptera
XP052863626.1	*Anopheles cruzii*	AncruNAGK	Diptera
XP034115884.1	*Drosophila albomicans*	DralbNAGK	Diptera
XP017067764.2	*Drosophila eugracilis*	DreugNAGK	Diptera
XP001993809.1	*Drosophila grimshawi*	DrgriNAGK	Diptera
BFF90860.1	*Drosophila madeirensis*	DrmadNAGK	Diptera
XP043864141.1	*Drosophila mojavensis*	DrmojNAGK	Diptera
XP039495489.1	*Drosophila santomea*	DrsanNAGK	Diptera
XP065089659.1	*Ochlerotatus camptorhynchus*	OccamNAGK	Diptera
XP030369508.1	*Scaptodrosophila lebanonensis*	SclebNAGK	Diptera
XP055598868.1	*Uranotaenia lowii*	UrlowNAGK	Diptera
XP001950774.1	*Acyrthosiphon pisum*	AcpisNAGK	Hemiptera
KAF0768210.1	*Aphis craccivora*	ApcraNAGK	Hemiptera
XP027836960.2	*Aphis gossypii*	ApgosNAGK	Hemiptera
XP018908713.1	*Bemisia tabaci*	BetabNAGK	Hemiptera
XP015376913.1	*Diuraphis noxia*	DinoxNAGK	Hemiptera
XP025206365.1	*Melanaphis sacchari*	MesacNAGK	Hemiptera
XP060880873.1	*Metopolophium dirhodum*	MedirNAGK	Hemiptera
XP022162754.1	*Myzus persicae*	MyperNAGK	Hemiptera
XP026810213.1	*Rhopalosiphum maidis*	RhmaiNAGK	Hemiptera
XP060839872.1	*Rhopalosiphum padi*	RhpadNAGK	Hemiptera
XP025406246.1	*Sipha flava*	SiflaNAGK	Hemiptera
XP018044326.1	*Atta colombica*	AtcolNAGK	Hymenoptera
XP050594253.1	*Bombus affinis*	BoaffNAGK	Hymenoptera
XP050484304.1	*Bombus huntii*	BohunNAGK	Hymenoptera
XP025262455.1	*Camponotus floridanus*	CafloNAGK	Hymenoptera
XP050449384.1	*Cataglyphis hispanica*	CahisNAGK	Hymenoptera
XP011309990.1	*Fopius arisanus*	FoariNAGK	Hymenoptera
XP043521026.1	*Frieseomelitta varia*	FrvarNAGK	Hymenoptera
XP032675250.1	*Odontomachus brunneus*	OdbruNAGK	Hymenoptera
XP015188838.1	*Polistes dominula*	PodomNAGK	Hymenoptera
XP018370857.1	*Trachymyrmex cornetzi*	TrcorNAGK	Hymenoptera
XP046994663.1	*Schistocerca americana*	ScameNAGK-1	Orthoptera
XP046994664.1	*Schistocerca americana*	ScameNAGK-2	Orthoptera
XP049777890.1	*Schistocerca cancellata*	SccanNAGK-1	Orthoptera
XP049777889.1	*Schistocerca cancellata*	SccanNAGK-2	Orthoptera
XP049777895.1	*Schistocerca cancellata*	SccanNAGK-3	Orthoptera
XP049862897.1	*Schistocerca gregaria*	ScgreNAGK	Orthoptera
XP049810707.1	*Schistocerca nitens*	ScnitNAGK-1	Orthoptera
XP049810706.1	*Schistocerca nitens*	ScnitNAGK-2	Orthoptera

XP047113104.1	*Schistocerca piceifrons*	ScpicNAGK-1	Orthoptera
XP047113106.1	*Schistocerca piceifrons*	ScpicNAGK-2	Orthoptera

Table S4. Information of insect UAP proteins.

Accession number	Species	Gene Name	Order
QWX20093.1	*Henosepilachna vigintioctopunctata*	HvUAP-3	Coleoptera
XP001164534.1	*Tribolium castaneum*	TcUAP-1	Coleoptera
XP066247072.1	*Euwallacea similis*	EsUAP-2	Coleoptera
XP066155390.1	*Euwallacea fornicatus*	EfUAP	Coleoptera
WDW19220.1	*Holotrichia parallela*	HpUAP	Coleoptera
XP001164533.1	*Tribolium castaneum*	TcUAP-2	Coleoptera
XP065165545.1	*Dalotia coriaria*	DcUAP-2	Coleoptera
XP016945538.2	*Drosophila suzukii*	DsUAP-1	Diptera
XP016945540.2	*Drosophila suzukii*	DsUAP-2	Diptera
XP065358398.1	*Calliphora vicina*	CvUAP	Diptera
XP064546920.1	*Drosophila montana*	DmUAP	Diptera
ADD18880.1	*Glossina morsitans*	GmUAP	Diptera
AGE89783.1	*Bactrocera dorsalis*	BdUAP	Diptera
XP067620802.1	*Eurosta solidaginis*	EsUAP-1	Diptera
JAB57776.1	*Corethrella appendiculata*	CaUAP	Diptera
XP065080006.1	*Ochlerotatus camptorhynchus*	OcUAP	Diptera
AAU25808.1	*Aedes aegypti*	AaUAP	Diptera
JAV27201.1	*Culex tarsalis*	CtUAP	Diptera
KAG8322854.1	*Homalodisca vitripennis*	HvUAP-1	Hemiptera
JAP04341.1	*Triatoma dimidiata*	TdUAP	Hemiptera
JAC16023.1	*Triatoma infestans*	TiUAP	Hemiptera
JAW09782.1	*Panstrongylus lignarius*	PlUAP	Hemiptera
XP014289230.1	*Halyomorpha halys*	HhUAP	Hemiptera
AUX15121.1	*Sogatella furcifera*	SfUAP-1	Hemiptera
AQS60688.1	*Sogatella furcifera*	SfUAP-2	Hemiptera
XP018902053.1	*Bemisia tabaci*	BtUAP	Hemiptera
WPA94609.1	*Cacopsylla chinensis*	CcUAP-1	Hemiptera
WEV88950.1	*Diaphorina citri*	DcUAP-1	Hemiptera
XP065199959.1	*Planococcus citri*	PcUAP	Hemiptera
WPA94608.1	*Cacopsylla chinensis*	CcUAP-2	Hemiptera
XP066595137.1	*Prorops nasuta*	PnUAP	Hymenoptera
XP067211017.1	*Linepithema humile*	LhUAP	Hymenoptera
XP043272416.1	*Venturia canescens*	VcUAP	Hymenoptera
XP034935842.1	*Chelonus insularis*	CiUAP	Hymenoptera
XP011305910.1	*Fopius arisanus*	FaUAP	Hymenoptera
ACN29686.1	*Spodoptera exigua*	SeUAP	Lepidoptera
WYX07335.1	*Helicoverpa armigera*	HaUAP	Lepidoptera
WQQ44657.1	*Mythimna separata*	MsUAP	Lepidoptera
WIV69443.1	*Hyphantria cunea*	HcUAP	Lepidoptera

AZM68712.1	*Heortia vitessoides*	HvUAP-2	Lepidoptera
QTU76246.1	*Cnaphalocrocis medinalis*	CmUAP-1	Lepidoptera
AKO90063.1	*Cnaphalocrocis medinalis*	CmUAP-2	Lepidoptera
QIJ96704.1	*Glyphodes pyloalis*	GpUAP	Lepidoptera
KOB71416.1	*Operophtera brumata*	ObUAP	Lepidoptera
XP001296486.1	*Bombyx mori*	BmUAP	Lepidoptera

Table S5. Information of insect PAGM proteins.

**Accession number**	Species	**Gene Name**	**Order**
XP018318404.1	*Agrilus planipennis*	AplPAGM1	Coleoptera
XP018318405.1	*Agrilus planipennis*	AplPAGM2	Coleoptera
XP019881850.1	*Aethina tumida*	AtuPAGM	Coleoptera
XP044751813.1	*Coccinella septempunctata*	CsePAGM	Coleoptera
XP017769387.1	*Nicrophorus vespilloides*	NvePAGM	Coleoptera
XP022917833.2	*Onthophagus taurus*	OtaPAGM	Coleoptera
XP034103246.1	*Drosophila albomicans*	DalPAGM	Diptera
XP017861382.1	*Drosophila arizonae*	DarPAGM	Diptera
XP017128684.1	*Drosophila elegans*	DelPAGM	Diptera
XP039487418.1	*Drosophila santomea*	DsaPAGM	Diptera
XP067646195.1	*Eurosta solidaginis*	EsoPAGM	Diptera
XP030375425.1	*Scaptodrosophila lebanonensis*	SlePAGM	Diptera
XP018910428.1	*Bemisia tabaci*	BtaPAGM	Hemiptera
XP014242302.1	*Cimex lectularius*	ClePAGM	Hemiptera
XP050535514.1	*Daktulosphaira vitifoliae*	DviPAGM	Hemiptera
XP054258516.1	*Macrosteles quadrilineatus*	MquPAGM	Hemiptera
XP039275739.1	*Nilaparvata lugens*	NluPAGM	Hemiptera
XP060839221.1	*Rhopalosiphum padi*	RpaPAGM	Hemiptera
XP025421379.1	*Sipha flava*	SflPAGM	Hemiptera
XP011061263.1	*Acromyrmex echinatior*	AecPAGM	Hymenoptera
XP043791281.1	*Apis laboriosa*	AlaPAGM	Hymenoptera
XP050578959.1	*Bombus affinis*	BafPAGM	Hymenoptera
XP017756535.1	*Eufriesea mexicana*	EmePAGM	Hymenoptera
XP032679390.1	*Odontomachus brunneus*	ObrPAGM	Hymenoptera
XP018365045.1	*Trachymyrmex cornetzi*	TcoPAGM	Hymenoptera
XP063369199.1	*Cydia amplana*	CamPAGM	Lepidoptera
XP063629719.1	*Cydia splendana*	CspPAGM	Lepidoptera
XP069363129.1	*Maniola hyperantus*	MhyPAGM	Lepidoptera
XP050357517.1	*Nymphalis io*	NioPAGM	Lepidoptera
XP045539925.1	*Papilio machaon*	PmaPAGM	Lepidoptera

XP047539705.1	*Vanessa atalanta*	VatPAGM	Lepidoptera
XP066998594.2	*Anabrus simplex*	AsiPAGM1	Orthoptera
XP066998595.2	*Anabrus simplex*	AsiPAGM2	Orthoptera
XP046995029.1	*Schistocerca americana*	SamPAGM	Orthoptera
XP049807680.1	*Schistocerca nitens*	SniPAGM	Orthoptera
XP047113091.1	*Schistocerca piceifrons*	SpiPAGM	Orthoptera
XP049956359.1	*Schistocerca serialis cubense*	SsePAGM	Orthoptera

Table S6. Information of insect GNA proteins.

Accession number	Species	Gene Name	Order
XP019771203.2	*Dendroctonus ponderosae*	DpoGNA	Coleoptera
XP045478570.1	*Harmonia axyridis*	HaxGNA1	Coleoptera
XP045478571.1	*Harmonia axyridis*	HaxGNA2	Coleoptera
XP030763427.1	*Sitophilus oryzae*	SorGNA1	Coleoptera
XP030763428.1	*Sitophilus oryzae*	SorGNA2	Coleoptera
XP063919344.1	*Zophobas morio*	ZmoGNA	Coleoptera
XP052868291.1	*Anopheles cruzii*	AcrGNA	Diptera
XP052900220.1	*Anopheles moucheti*	AmoGNA	Diptera
XP020713733.1	*Ceratitis capitata*	CcaGNA	Diptera
XP017967416.1	*Drosophila navojoa*	DnaGNA	Diptera
XP032578158.1	*Drosophila sechellia*	DseGNA	Diptera
XP011185974.1	*Zeugodacus cucurbitae*	ZcuGNA	Diptera
XP018904263.1	*Bemisia tabaci*	BtaGNA	Hemiptera
JAG43038.1	*Lygus hesperus*	LheGNA1	Hemiptera
JAQ06590.1	*Lygus hesperus*	LheGNA2	Hemiptera
XP054257661.1	*Macrosteles quadrilineatus*	MquGNA1	Hemiptera
XP054257662.1	*Macrosteles quadrilineatus*	MquGNA2	Hemiptera
XP054257663.1	*Macrosteles quadrilineatus*	MquGNA3	Hemiptera
XP025418201.1	*Sipha flava*	SflGNA	Hemiptera
PBC33923.1	*Apis cerana*	AceGNA	Hymenoptera
XP018407595.1	*Cyphomyrmex costatus*	CcoGNA	Hymenoptera
XP014480235.1	*Dinoponera quadriceps*	DquGNA	Hymenoptera
XP015181299.1	*Polistes dominula*	PdoGNA	Hymenoptera
XP011882165.1	*Vollenhovia emeryi*	VemGNA	Hymenoptera
XP047354208.1	*Vespa velutina*	VveGNA	Hymenoptera
XP028038668.1	*Bombyx mandarina*	BmaGNA	Lepidoptera
NP001040128.1	*Bombyx mori*	BmoGNA	Lepidoptera
XP061721730.1	*Cydia pomonella*	CpoGNA	Lepidoptera
XP053614140.1	*Plodia interpunctella*	PinGNA	Lepidoptera
XP013139244.1	*Papilio polytes*	PpoGNA	Lepidoptera
XP047536742.1	*Vanessa atalanta*	VatGNA	Lepidoptera
XP046992170.1	*Schistocerca americana*	SamGNA	Orthoptera

XP049777764.1	*Schistocerca cancellata*	ScaGNA	Orthoptera
XP049858254.1	*Schistocerca gregaria*	SgrGNA	Orthoptera
XP049788656.1	*Schistocerca nitens*	SniGNA1	Orthoptera
XP049788657.1	*Schistocerca nitens*	SniGNA2	Orthoptera
XP047113998.1	*Schistocerca piceifrons*	SpiGNA	Orthoptera

Table S7. Design of 300-bp dsGKP template

Sequcence dsGKP template	5'CTACGAGAACGACCCGATGAAGCTCCTGGCCTCGCTCGGGGTG<br>GACGCGACCCCGGCGACGCGGAGGCTGGTGCTGGGCGCCGAGG<br>CGGCGCTGTGGACGATCACTCTTCAATGGCAAAGGTGAAAGACT<br>GTCGGAGCTAACTGGAGTTGGTACAAATTACCTGACCCTAGGAAT<br>GGATGTTGTACACGATAGGATAAATAATGGCAAGTTCCAAGTTGT<br>AGAATATAGTGAAATAACTTTGGAAACAGCTATGAAACAAGATGA<br>TGATGGTAAATTAACTTTCAGGGCAGGGAGCATTT3'

Note: The first (underscored), second, and third (underscored) 100-bp fragments of GKP are derived from *BtNAG1* (XM019048003), *BtNAGK* (XM019053168), and *BtUAP* (XM019046508), respectively.

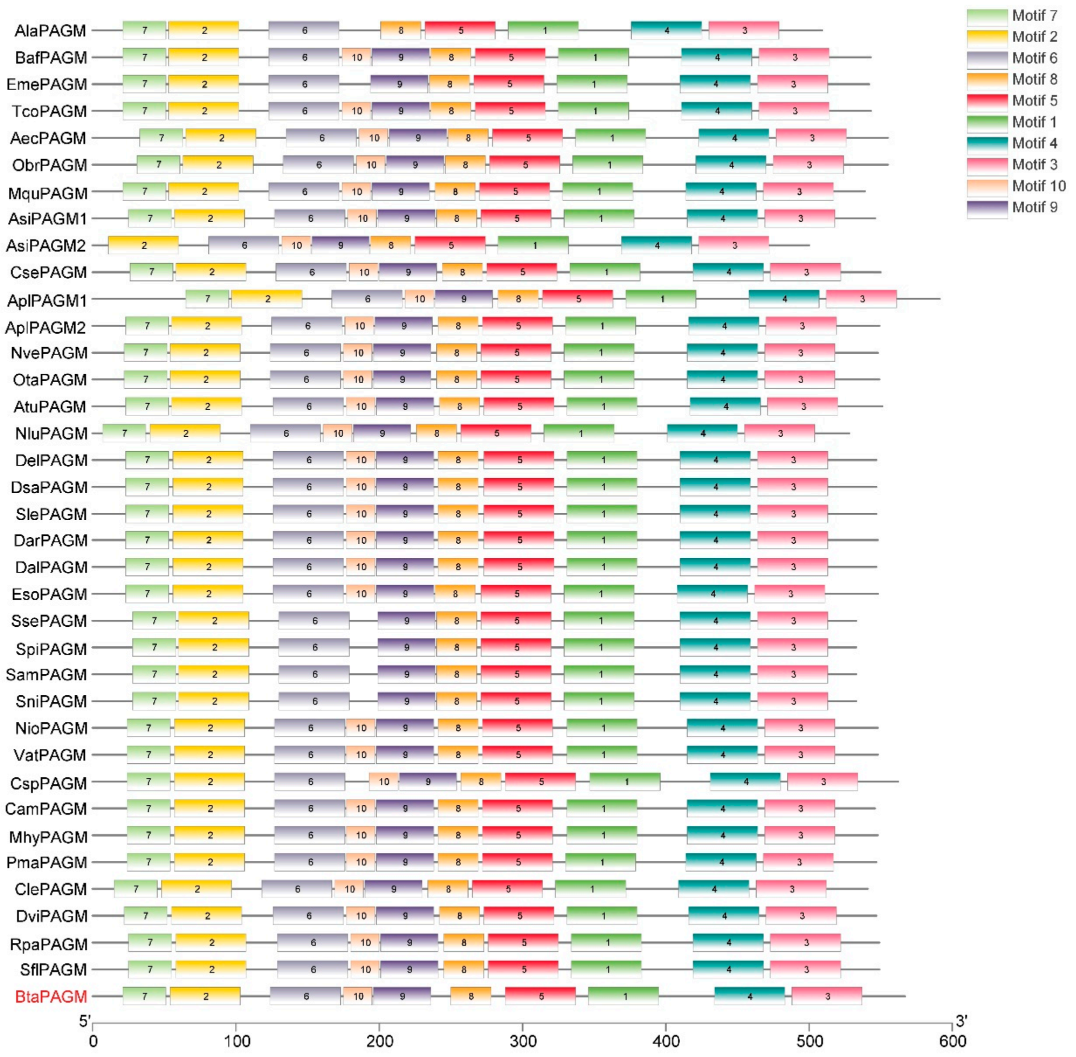

Figure S1. Analysis of conserved motifs in insect PAGM proteins. The x-axis indicates the number of amino acid residues. The *Bemisia tabaci* PAGM protein (BtPAGM) is highlighted in red. The analysis was done on the MEME suit server (https://meme-suite.org/meme/ (assessed accessed on 15 October 2023)). The detailed information of the 37 sequences used can be found in Table S4.
